# Supporting GPs and people with hypertension to maximise medication use to control blood pressure: Protocol for a pilot cluster RCT of the MIAMI intervention

**DOI:** 10.12688/hrbopenres.13661.1

**Published:** 2023-01-17

**Authors:** Eimear Morrissey, Andrew Murphy, Patrick Murphy, Louise O'Grady, Molly Byrne, Monica Casey, Eamon Dolan, Sinead Duane, Hannah Durand, Paddy Gillespie, Peter Hayes, Anna Hobbins, Lisa Hynes, John William McEvoy, John Newell, MIAMI PPI group, Gerard Molloy

**Affiliations:** 1 School of Psychology, University of Galway, Galway, Ireland; 2 HRB Primary Care Clinical Trials Network, University of Galway, Galway, Ireland; 3 School of Medicine, University of Limerick, Limerick, Ireland; 4 Connolly Hospital, Blanchardstown, Dublin, Ireland; 5 J.E. Cairnes School of Business and Economics, University of Galway, Galway, Ireland; 6 HRB Trials Methodology Research Network, University of Galway, Galway, Ireland; 7 Division of Psychology, Faculty of Natural Sciences, University of Stirling, Stirling, UK; 8 Health Economics and Policy Analysis Centre, Institute for Lifecourse and Society, University of Galway, Galway, Ireland; 9 CURAM, SFI Research Centre for Medical Devices (13/RC/2073_P2), University of Galway, Galway, Ireland; 10 Croí, The West of Ireland Cardiac and Stroke Foundation, Galway, Ireland; 11 National Institute for Prevention and Cardiovascular Health, University of Galway, Galway, Ireland; 12 School of Mathematical and Statistical Sciences, University of Galway, Galway, Ireland

**Keywords:** Hypertension, primary care, adherence, feasibility, protocol

## Abstract

**Background:** Hypertension is one of the most important risk factors for stroke and heart disease. Recent international guidelines have stated that *'poor adherence to treatment – in addition to physician inertia - is the most important cause of poor blood pressure control'.* The MaxImising Adherence, Minimising Inertia (MIAMI) intervention, which has been developed using a systematic, theoretical, user-centred approach, aims to support general practitioners (GPs) and people with hypertension to maximise medication use, through the facilitation of adequate information exchange within consultations about long-term antihypertensive medication use and adherence skill development. The aim of the MIAMI pilot cluster randomised controlled trial (RCT) is to gather and analyse feasibility data to allow us to (1) refine the intervention, and (2) determine the feasibility of a definitive RCT.

**Methods:** GP practices (n = 6) will be recruited and randomised to the intervention arm (n = 3) or usual care control arm (n = 3). Each practice will recruit 10 patient participants. For a patient to be eligible they must have a diagnosis of hypertension, be on two or more anti-hypertensive medications, must not be achieving recommended blood pressure levels, and be over the age of 65 years. Participants in the intervention arm will meet their GP and receive the MIAMI intervention twice over three months. Quantitative data collection will take place at baseline and three month follow up. A pilot health economic analysis and a qualitative sub-study will also be incorporated into the study.

**Discussion:** This pilot cluster RCT of the MIAMI intervention will allow us to gather valuable acceptability and feasibility data to further refine the intervention so it optimally designed for both GP and patient use. In particular, the qualitative component will provide an insight into GP and patient experiences of using the intervention.

## Introduction

Hypertension is one of the most important risk factors for stroke and heart disease ^1^. A landmark study of twelve high income countries from 1976–2017 concluded that hypertension ‘ *control rates have plateaued in the past decade, at levels lower than those in high quality hypertension programmes*’ ^1^. International comparisons suggests that in Ireland there are relatively low levels of awareness of hypertension and relatively poor levels of control and suboptimal treatment. For example, estimates from the same international study indicate that the proportion of Irish male patients with controlled blood pressure is 17%, while the comparable figure in Canada is 69% ^1^. A parallel paper reviewing the state of hypertension care for 1.1 million adults in 44 low- and middle-income countries ^2^ identified countries in each world region that performed better than expected from their economic development. Many of these countries, which spend much less on healthcare than Ireland, significantly outperformed Ireland in hypertension awareness, treatment and control.

Recent international guidelines have stated that *'poor adherence to treatment – in addition to physician inertia - is the most important cause of poor blood pressure (BP) control'* ^3^. The adverse impact of poor adherence to anti-hypertensive medication has been recently shown in a Korean study ^4^. This showed, in a random sample of 33,728 patients with hypertension, that poor medication adherence was associated with higher mortality and the development of key negative cardiovascular outcomes. For example, patients with poor medication adherence had, in comparison to those with good adherence, worse ischaemic heart disease mortality (hazard ratio [HR] 1.64; 95% confidence interval [CI] 1.16-2.31) and cerebral haemorrhage (HR 2.19; 95% CI 1.28-3.77). Improving adherence to antihypertensive medication has the potential to quickly lower blood pressure and to reduce the related cardiovascular risk in most patients ^3^. Gupta and colleagues in a non-randomised study of 238 UK patients with apparent resistant hypertension (high blood pressure that is not responsive to treatment) attending a tertiary referral centre utilised sequential biochemical urine screening for assessment of adherence ^5^. The results of this testing were fed back to patients, with subsequent significant improvements in both adherence and blood pressure (systolic 32.6mmHg and diastolic 7.5mmHg). This approach, referred to as chemical adherence testing, may have some potential to enhance collaboration between patients and physicians in optimising the management of hypertension ^6^.

Clinical inertia is largely seen as failure to advance therapy when appropriate to do so, and this is particularly relevant in the treatment of hypertension. Two Irish studies have confirmed that suboptimal dosing is a significant issue in Irish general practice populations of patients with resistant hypertension ^7^ and post stroke and/or transient ischaemic attack ^8^. In 646 patients with apparent resistant hypertension from 16 independent centres, i.e., community-based general practice sites, 19% of patients had adequate dosing for each hypertensive medication according to the World Health Organisation defined daily dose guidelines ^9^. However, in terms of the definition of ‘clinical inertia,’ it can be argued that failure to advance therapy when indicated is just one facet of a larger phenomenon. Khunti and Davis argue that ‘clinical inertia’ is not only related to escalation of therapy but is a much wider concept and encompasses failure to improve care at many levels of health care ^10^. In the context of hypertension care, we know that conversations around medication adherence are often avoided. A recent survey of 200 hypertension specialists in 30 countries found that the topic of medication taking was under attended to, despite its importance ^11^. A similar survey of healthcare professionals (n = 3196) working in primary care in Europe found that less than half of respondents ever asked their patients whether they have missed any doses of their medication ^12^. It can be argued, therefore, that tackling clinical inertia in hypertension care involves addressing issues of medication adherence as well as optimal dosing.

The ‘MaxImising Adherence, Minimising Inertia’ (MIAMI) intervention, which has been developed using a systematic, theoretical, user-centred approach, aims to support GPs and people with hypertension to maximise medication use. The Behaviour Change Wheel ^13^ and Collective Intelligence methodology ^14^ were used in development and a detailed description of the process will be described elsewhere. A full description of the intervention can be seen in Table 2. The MIAMI intervention specifically targets older patients (over 65 years of age), as they are at increased risk from both hypertension ^3^ and medication non-adherence, given the increased likelihood of multimorbidity and the related challenge of polypharmacy in older adulthood ^15, 16^.

### Aims of the MIAMI cluster pilot RCT

The aim of the MIAMI pilot cluster randomised controlled trial (RCT) is to gather and analyse feasibility data to allow us to (1) refine the MIAMI intervention, and (2) determine the feasibility of a definitive RCT.

Specifically, the MIAMI pilot cluster RCT has the following objectives:

1. To investigate if the MIAMI intervention is acceptable to GPs and people with hypertension;
2. To collect pilot qualitative and quantitative data to assess the feasibility of recruitment and retention of both practices and patients;
3. To collect pilot qualitative and quantitative data to assess the feasibility of outcomes and measures used;
4. To conduct a pilot health economic assessment of the MIAMI intervention;
5. To inform the sample size calculation, including the optimal number of GP practices (clusters) and people with hypertension (participants), for a definitive cluster RCT;
6. To collect pilot quantitative and qualitative data to assess the feasibility of a ‘Study Within A Trial’ focused on the impact of an informational video on study retention levels

This protocol was submitted to ISRCTN on 6 ^th^ October 2022 (awaiting approval) and all study materials can be found as *Extended data* ^17^.

### Design

This is a pilot cluster RCT with an intervention arm and a control arm. The intervention arm is the MIAMI intervention and the control arm is usual care.

### Inclusion and exclusion criteria

Please see Table 1 for inclusion and exclusion criteria.

**Table 1.** Inclusion and exclusion criteria.

Inclusion criteria for GP practices	Inclusion criteria for GP participants	Inclusion criteria for patient participants
• Within catchment area of biochemistry lab at University Hospital Galway • Use Socrates software system	• Doctors who are providing patient care in the practice	• A confirmed diagnosis of hypertension • Participants must be over the age of 65 • Participants must be on two or more anti- hypertensive medications • Participants must not be achieving recommended blood pressure levels, i.e., clinic readings are higher than 140/90 mmHg or day ABPM 135/ 85 mmHg • In the judgement of the GP regarding a change in medication, the balance of risk / benefit lies in favour of benefit.
		**Exclusion criteria for patient participants**
		• Inability to give informed consent • Resident in a nursing home • Currently attending a hospital resistant hypertension clinic

**Table 2.** MIAMI intervention.

GP intervention components		
Component	Details	Timing of delivery
Training videos	30 minute training (6 short videos and quiz) Part 1: Adherence to medication, including the extent of non-adherence, key factors associated with adherence, and types of non-adherence Part 2: Three case studies to illustrate how you might support patients’ medication adherence in different scenarios Part 3: Skills and strategies to support adherence Part 4: A case study illustrating the use of the MIAMI intervention	Beginning of study, before meeting any patient participants
MIAMI booklet	Booklet contains • Blood pressure thresholds (American College of Cardiology) • Oral anti-hypertensive drugs (American College of Cardiology) • Core drug treatment strategy for uncomplicated hypertension (European College of Cardiology) • List of available single pill combinations • Simple reiteration of key training messages	Beginning of study, before meeting any patient participants
Drop down menu on computer to guide consultation* * *All GP practices in this study will be * *using Socrates software. Dropdown * *menus are easily integrated into this * *software and will be done by the * *research team prior to study beginning.*	Drop down menu on computer which contains a guide to the consultation. • Discussed ABPM YES/NO • Discussed urine results YES/NO • Made an agreed plan which may include: single pill combinations, ‘blister pack’ specification: YES/NO • Asked patient to form a habit (pair medication taking with another stable behaviour e.g. a mealtime/brushing teeth/ Coronation Street): YES/NO • Wrote out the agreed plan on the ‘MIAMI pre-consultation tool’ YES/NP • Sent relevant text message: YES/NO Text to copy and paste into text messages You can set up text messages to remind you to take your medication at dontforget.ie Information about blood pressure and blood pressure medication is available on the Croí website https://croi.ie/health/heart-conditions/ high-blood-pressure/	Set up prior to study beginning. To be used during consultations.
**Patient intervention components**		
**Component**	**Details**	**Timing of delivery**
Pre-consultation plan	Short document containing • ‘What do you want to talk about at your blood pressure appointment today’ (to be filled in prior to consultation) • Textbox for BP reading and whether it is in target • Textbox for goal setting and action planning (to be used during consultation)	Given at practice visit 2, prior to GP consultation. To be used at the beginning and during the consultation. This is optional and patient participants can decide whether or not they want to use it.
GP consultation	Discussion of ABPM and urine test results. Use of pre-consultation plan to create shared action plan GP to review prescription and adjust to include single pill combinations and blister pack if appropriate GP to advise on habit creation GP to send text messages on dontforget.ie and Croí website if appropriate	GP consultation
Croí website	Page on website containing (both text and video format) • information on BP • BP values • how medication works • benefits of medication possible side effects	GP to recommend to patient during consultation

### Sample size calculations

As this is a pilot study, a formal sample size calculation is not warranted. One of the aims of the study is to generate the estimates needed for a sample size calculation for a future definitive trial. In order to generate the reliable estimates needed, it is proposed that six clusters (three in the intervention arm, three in the control arm) each containing 10 patient participants are recruited. The number of clusters chosen is governed by pragmatism and to provide a representative sample (using best judgement) of the diversity in the population of clusters from which data in the definitive trial will be sampled. 

### Recruitment

***GP practice recruitment***

General practices affiliated with the HRB Primary Care Clinical Trials Network Ireland, who have previously expressed an interest in hypertension research and are within the catchment area of the biochemistry laboratory at University Hospital Galway, will be invited to participate. A detailed practice information sheet will be provided (see *Extended data*). If invited practices express interest in taking part, research staff on the project will follow up with a telephone call to provide further details about the study. All participating practices will be offered €100 per recruited patient participant to cover the additional administration costs, as well as a right to keep a 24-hour ambulatory blood pressure monitor (ABPM) after the study has ended.

***Patient participant recruitment***

The practice administrator/manager, supported by the research team, will search the practice records using the inclusion and exclusion criteria to identify eligible patient participants.

Once identified, the practice will post a letter of invitation and information sheet to a random selection of 20 patients from each practice (this is based on a 50% recruitment rate as seen in a previous feasibility study ^18^). The random selection will be facilitated using random.org. These patients will be asked to contact the research team if interested in taking part. If there is no response after 10 days, the practice will contact eligible patients by phone and explain the information in the letters and offer an opportunity to ask questions. If the target of 10 participants is not met at that point, another 10- 20 letters will be sent out, dependent on the response to date. This will continue until the target is met. All interested patients will receive a consent form in the post, which they will be asked to return to the team before the trial begins. All patient participants will be offered a €40 multi-store voucher to cover costs associated with their participation (e.g., travel to the practice for study visits).

### Randomisation procedures

After all participants have been recruited, three GP practices will be randomised to the MIAMI intervention and three will be randomised to the usual care control. The code to generate the randomisation plan will be written in R (using a reproducible random generation seed) and implemented by an independent statistician.

### Intervention procedures

The MIAMI intervention will be delivered at a minimum of one GP appointment during a three-month period. The intervention arm and control arm are described below.

***MIAMI intervention arm***

The MIAMI intervention is a structured set of supports for GPs and patients with hypertension to facilitate adequate information exchange within consultations about long-term antihypertensive medication use and adherence skill development. Full details are in Table 2. It will include the following, where indicated:

Patients

- Wearing an ABPM and providing a urine sample for a chemical urine test of adherence.
- Filling out a brief ‘pre-consultation plan’ before a GP consultation.

GPs

- Training in how to structure a consultation around adherence issues. This will be provided through online videos and will take a maximum of 30 minutes.
- A booklet with key takeaways from training videos and current guidance around blood pressure medication prescribing (from the European Society of Hypertension ^3^ and American College of Cardiology ^19^)
- A drop down menu on GP software which will provide a reminder of consultation guide and a list of resources that can be recommended to the patient (e.g., medication reminders - dontforget.ie and BP information - croi.ie/health/heart-conditions/high-blood-pressure/)

**Figure 1.**
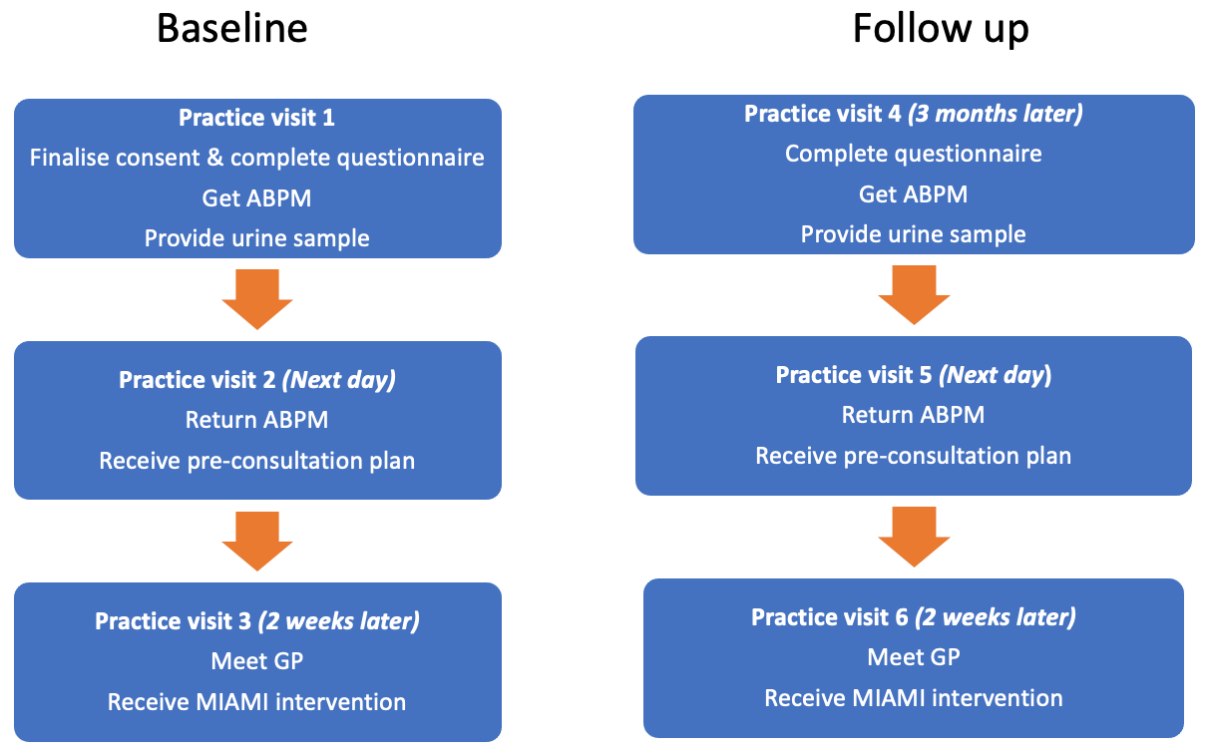
Patient flow through MIAMI intervention arm.

The patient flow through the intervention arm can be seen in Figure 1.

***Control group***

Participants in the control group will receive usual care. This would involve visits by patients to GPs where blood pressure would be measured manually via office-based sphygmomanometer or automated blood pressure device and management decision on how best to proceed would stem from this meeting, which is typically scheduled every 3-4 months for patients over 65. Blood pressure may also be assessed via out of office assessment if manual readings are high. Previous research has demonstrated typical appointments between hypertensive patient and their GP does not often involve structured discussions about adherence ^11, 12^.

**Figure 2.**
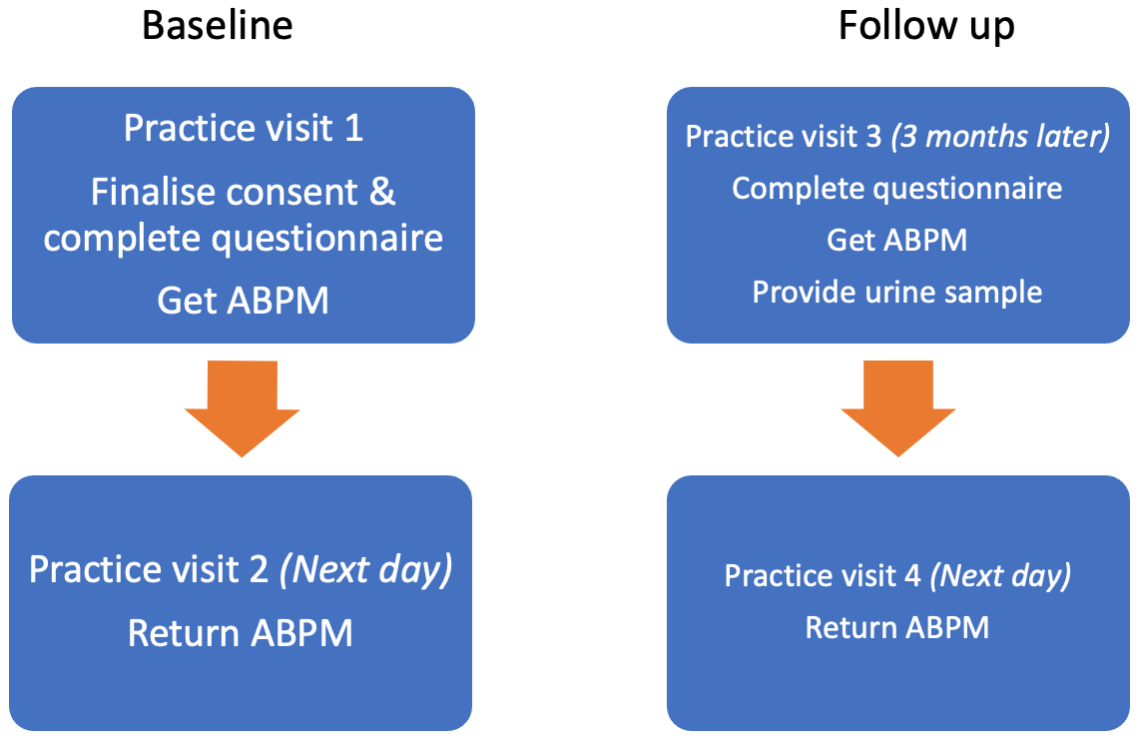
Patient flow through the control arm.

Patient flow through the control arm can be seen in Figure 2.

### Data collection

***Participant quantitative data collection***

*Patients*

Patient data collection will occur at two time points during the trial. These include:

T1) Baseline

T2) Follow up (3 months later)

Self-report questionnaires for T1 and T2 will be administered to participants at the GP practice. The questionnaire will be paper based and will include questions on demographics, outcome measures, and resource use (for health economic analysis). For the blood pressure measurement outcomes, participants will be given an ABPM at T1 and T2. For the objective adherence outcome, participants will provide a urine sample which will undergo chemical adherence testing ^6^. All other quantitative data will be taken from practice records by the lead researcher.

Please see Table 3 for description of outcome measures.

**Table 3.** Patient outcome measures.

Outcome	Measurement	When	Method of capture
Systolic BP	24 hour ABPM (average day reading)	T1 & T2	Researcher entry
Diastolic BP	24 hour ABPM (average day reading)	T1 & T2	Researcher entry
BP control	24 hour ABPM (readings above or below 140/90 mmHg)	T1 & T2	Researcher entry
Medication adherence	Urine screen (Adherence ratio *)	(T1) & T2 **	Chart review
Medication adherence	Prescription refill record	T1 & T2	Chart review
Medication adherence	Medication Adherence Report Scale ^23^	T1 & T2	Patient self- report
Beliefs about medication	Beliefs about Medicines Questionnaire ^24^ Illness Perceptions Questionnaire– Revised (‘treatment control’ & ‘consequences’ items) ^25^	T1 & T2	Patient self- report
Habit strength	Self-Report Behavioural Automaticity Index ^26^	T1 & T2	Patient self-report
Patient assessment of care	Patient Assessment of Chronic Illness Care ^27^ ***	T1 & T2	Patient self-report
Medication prescription/pill burden	Type and dosage	T1 & T2	Chart review
Health related QoL	EuroQoL-5D-5L	T1 & T2	Patient self- report
Wellbeing	ICECAP-O	T1 & T2	Patient self- report

** *Ratios are calculated for each patient – based on the medications that could be detected – by dividing the total number of antihypertensive medications detected in spot urine into the total number of detectable antihypertensive medications prescribed, whereby total non-adherence was equal to 0 and perfect adherence was equal to 1* * *This outcome will not be captured at baseline in the control arm, due to a concern around the provision of clinical information to GPs without guidance on management. It will be captured at follow up in the control arm, as all GPs will have access to intervention materials at study end.* *** Four of the five subscales - recent psychometric evaluation evidence that indicates that ‘Follow-up/Coordination’ sub-scale and item 10 is the least psychometrically robust and that a 14-item measure with an underlying four factor structure provides more psychometrically robust measurement than the original 20 item scale with a 5 factor structure ^28^

***GPs***

This data will be collected at T1 only. A questionnaire will gather demographic and personal information including: length of time working in primary care, employment basis (full-time, part-time, and other), practice location (urban/rural) and practice size.

***Feasibility and acceptability data***

The following will also be measured:

1. Recruitment of GP practices will be assessed by documenting the number of invitations sent, the number of refusals and number of acceptances.
2. Recruitment of patients will be assessed by documenting the number of invitations sent, the number of initial responses, the number of follow-up phone-calls required, the number of refusals and the number of acceptances.
3. Attrition of participants will be documented at every time-point.
4. Levels of missing data in returned questionnaires will be reported.
5. The comprehensibility and acceptability of all questionnaires will be measured by asking participants how the questionnaires might be improved, and how long they took to complete.

***Qualitative evaluation of feasibility and acceptability***

A descriptive qualitative approach ^19^ will be used to explore the perceptions and experiences of people with hypertension (n = 6) and GPs (n = 3) of participating in the MIAMI study and their views as to the acceptability of the intervention. In the intervention arm, six patient participants will be invited to participate in one-to-one interviews at month 1 (post first GP visit) and again at month 3 (post follow up visit). These longitudinal interviews will give an insight into participants’ views and changes in perceptions as they progress through the study. In addition, GPs delivering the intervention (n = 3) will be interviewed at month 3. These interviews will focus on their experiences of delivering the intervention and their views on its acceptability and feasibility in practice.

Patient participants (n = 6) and GPs (n = 3) in the control arm will be invited to one-to-one interviews at month 3. The focus of these interviews will be to acceptability of taking part in a pilot RCT in the control arm. The interview guides can be seen as *Extended data*. Thematic analysis as outlined by Braun & Clarke ^20^ will be used to analyse the data. Rigour will be maintained by adhering to the criteria described by Lincoln & Guba ^21^ and the computer software package NVivo will be used to assist the analysis process.

***Fidelity***

Fidelity assessment will provide valuable information on the feasibility of intervention implementation, as well as information on the acceptability and usefulness of the fidelity assessment procedures which may require adaptation for the definitive RCT.

*MIAMI intervention training fidelity*

Attendance by GPs in each practice at the initial training method will be recorded. Analytics such as the number of clicks and time spent on each page will also be available from the GP training website.

Analytics such as number of clicks and time spent on each page will be available from the GP training website.

*MIAMI intervention delivery fidelity*

A checklist embedded as a ‘Drop-Down Menu’ in the Socrates practice software system will be developed for the distinct components in the MIAMI intervention. This checklist will be completed by GP participants during each consultation and subsequently reviewed by the research team.

Fidelity will also be explored in the qualitative interviews with patient and GP participants.

### Health economic analysis

A pilot health economic assessment of the MIAMI intervention will be conducted to provide preliminary cost effectiveness estimates and to inform the design of a future definitive economic evaluation. A healthcare perspective will be adopted with respect to costing. Data collection tools will be developed for the purposes of measuring data on resource use over the trial follow-up period. An exploratory process will be conducted to identify the resource use and costs associated with the delivery of the MIAMI intervention. Unit costs will be identified and applied to convert data on resource use to costs. For the pilot cost utility analysis, Quality Adjusted Life Years (QALYs) will be estimated using data collected using the EuroQol EQ-5D-5L instrument ^29^ and the ICECAP-O instrument ^30^ at baseline and follow up. A preliminary analysis will be undertaken to provide information on the incremental costs and incremental effects of the MIAMI intervention relative to the usual care control, and a range of techniques will be employed to address uncertainty. The pilot analysis is designed to determine the feasibility of the approaches adopted and not cost effectiveness.

### Statistical analysis

Suitable summary statistics (e.g., mean, standard deviation, and frequency) will be provided for the main outcomes. These summary statistics will guide the sample size calculation needed for the future definitive two arm cluster RCT ^31, 32^.

### Progression to a full RCT

The following pre-defined stop/go criteria in Table 4 will be used to inform the decision on whether to proceed to a full trial.

**Table 4.** Stop/go criteria.

	Go – proceed with full RCT	Amend – proceed to full RCT with changes	Stop – do not proceed unless major changes are possible
1. Feasibility of practice recruitment Can 6 practices be recruited to take part in 3 months?	If ≥5 practices are recruited to take part in 3 months	If ≥5 practices are recruited, but it takes longer than predicted (e.g. 3–6 months)	Unable to recruit at least 4 practices.
2. Feasibility of patient recruitment Can 10 patients per practice (total n = 60) be recruited?	If 8–10 patients are recruited in one month per practice: total of 60 (100%)	If 6-7 patients are recruited in one month per practice: total of 36–60 (60% to ≤100%)	If <6 patients are recruited in one month per practice: total of ≤36 (<60%)
3. Feasibility of practice retention Can all 6 practices be retained in the study until completion?	≥5 retained	≥4 retained	≤3 retained
4. Feasibility of patient retention * Can at least 90% of recruited patients be retained in the study until completion?	>9 patients (90%) retained	7–8 patients (70-80%) retained	<7 patients (<70%) retained
5. Intervention feasibility	Delivery of intervention judged strongly feasible by qualitative data	Delivery of intervention judged mainly feasible by qualitative data	Delivery of intervention judged problematic by qualitative data

*Retention will be defined as availability of final outcome measures.

The Decision-making after Pilot and feasibility Trials (ADePT) process involves examining 14 methodological issues that are pertinent to feasibility research. We will use this process, as well as the progression criteria, findings from the qualitative research, and discussions with the study research team, Trial Management Group, Trial Steering Committee and the public and patient involvement (PPI) panel to make a decision on whether to progress to full RCT.

### Study within a trial (SWAT)

A pilot SWAT (study within a trial) will be embedded within this pilot RCT. The objective of the SWAT will be to evaluate the effect of a video outlining the importance of the research on retention to the study. Videos have been used in the past to improve recruitment to studies ^33, 34^ and it is possible that this effect may also apply to retention. Half of the patient participants will be randomised to the intervention group, where at the mid-point of the study, they will receive a text message with a link to the video. The control group will not receive a text message and progress through the study as usual. The video will be less than five minutes long and contain information on the prevalence of high blood pressure, risks of high blood pressure, and justification for the current research. The primary outcome will be the number and proportion of participants who are retained in the study; however, as this is a pilot SWAT, we will not test for effectiveness. We will analyse the feasibility and acceptability of the SWAT through descriptive statistics and qualitative work.

### Public and patient involvement (PPI)

The study team includes a public and patient involvement (PPI) panel of six people living with hypertension. This panel meets quarterly and contributes to tasks such as development of the protocol, patient materials and SWAT materials. Two members of the panel also attend and contribute to Trial Steering Committee meetings.

## Ethics and dissemination

Ethical approval has been obtained for all intervention sites from the Irish College of General Practitioners on 23/8/22 (ICGP_REC_22_014). The results of this pilot cluster RCT will be published in a peer-reviewed journal.

## Study status

Five GPs practices have been recruited. Patient participant recruitment began in November 2022.

## Discussion

Supporting GPs and people living with hypertension to work together to maximise medication use has the potential to significantly lower blood pressure in primary care in Ireland. This pilot cluster RCT of the MIAMI intervention will allow us to gather valuable acceptability and feasibility data to further refine the intervention so it optimally designed for both GP and patient use. In particular, the qualitative component will provide an insight into GP and patient experiences of using the intervention.

The pilot cluster RCT will also allow us to the assess the potential feasibility issues involved in running a definitive RCT in the Irish primary care context. One possible difficulty that may arise are the logistics and safety of running such a study during flu season and the possibility of another wave of coronavirus disease 2019 (COVID-19) infection. As this pilot cluster RCT is due to begin in winter 2022, it should allow us to gain an insight into this context.

## Data Availability

No underlying data are associated with this article. Open Science Framework: MIAMI. https://doi.org/10.17605/OSF.IO/TYAR4 ^17^. This project contains the following extended data: Consent - GP.doc Consent - patient v2.docx Interview guides.docx Invite letter - GP v2.docx PIL - GP.doc PIL - patient.doc Questionnaire - GP.docx Questionnaire - patient.docx Data are available under the terms of the Creative Commons Zero "No rights reserved" data waiver (CC0 1.0 Public domain dedication).
