# Supplementary material for: Supporting GPs and people with hypertension to maximise medication use to control blood pressure: Protocol for a pilot cluster RCT of the MIAMI intervention
Source: HRB Open Res. 2023 Aug 21;6:6. Originally published 2023 Jan 17. [Version 2] doi: 10.12688/hrbopenres.13661.2 (PMC11109531; doi:10.12688/hrbopenres.13661.2)
Supplement: Peer review history

# Reviewer report: Reviewer response for version 2

**DOI:** 10.21956/hrbopenres.15086.r38415

**Published:** 2024-05-07

**Authors:** Peter Knapp

**Affiliations:** 1 University of York, York, England, UK

Hypertension is clearly a problem in Ireland but I think the authors overstate the extent of it. Their citation (1) has been referenced selectively. The data from those surveys show that the estimated prevalence of hypertension in Ireland was 43% in women and 56% in men. (The equivalent figures for Canada were 36% and 34%). The data for Ireland come from 2009-11 (since when the position may have improved) while the data for Canada are from 2016-17. Clearly there is a problem in Ireland (and the problem is relatively big compared to some other countries), but the issue has been reported selectively in the paper. 

 The details of the pilot trial design seem sensible, collecting both quantitative and qualitative data to inform the design of a further trial. However, I'm concerned by 2 of the design decisions:

 First, if clinical inertia is one source of problems, why is one of the patients' entry criteria taking at least two anti-hypertensives? Would an example of clinical inertia be not prescribing a 2nd medicine for patients whose hypertension was not responsive to the 1st medicine? Will this patient entry criterion potentially lead to exclusion of patients currently being treated with 'clinical inertia'? 

 Second, the authors describe two main sources of hypertension in older patients: clinical inertia (ie under-treatment) and patient non-compliance. While that seems convincing, the design of the trial potentially conflates the two sources of problems and might not allow them to be differentiated if the intervention is ineffective or only partly effective. Suppose the intervention has an effect on GPs' prescribing patterns but not on patient medicine-taking behaviour: a null result for the trial would not be an accurate reflection of the effect of the intervention. Can the data collection and analysis allow the two problem sources to be separated? 

Is the study design appropriate for the research question?

Partly

Is the rationale for, and objectives of, the study clearly described?

Yes

Are sufficient details of the methods provided to allow replication by others?

Yes

Are the datasets clearly presented in a useable and accessible format?

Not applicable

Reviewer Expertise:

Informaiton for patients; patient use of healthcare; trials; systematic reviews;

I confirm that I have read this submission and believe that I have an appropriate level of expertise to confirm that it is of an acceptable scientific standard, however I have significant reservations, as outlined above.

# Reviewer report: Reviewer response for version 2

**DOI:** 10.21956/hrbopenres.15086.r37038

**Published:** 2023-11-27

**Authors:** Sarah J Billups

**Affiliations:** 1 University of Colorado Health, Aurora, Colorado, USA

Interesting and methodical approach that may be generalizable to other populations if it proves effective, though the high frequency of patient visits may select for patients willing to comply with this schedule. 

 There are several typos throughout and some language that could use clarification (see below), but overall will be a valuable contribution to available evidence for the problems of medication adherence and inertia.

 Introduction:

 I realize this isn’t the point of the current study, but it should be noted that resistant hypertension has a more specific definition than is listed at the bottom of page 3, 1 ^st^ column.  See article by Yaxley ^1^

 In several places the language should be more precise, some examples:

 This statement is confusing: “19% of patients had adequate dosing … according to the World Health Organisation defined daily dose guidelines”  Adequate dosing is defined by the BP level, not an arbitrary dose.

 You state that  “…conversations around medication adherence are often avoided.”  I disagree with that term—overlooked perhaps, but to say avoided implies intent that is not evidence-based.

 You state that the MIAMI intervention has been developed “… to maximise medication use.”  I think you mean to optimize?

 You state: “The MIAMI intervention specifically targets older patients … increased risk from both hypertension and medication non-adherence, given increased likelihood of multimorbidity…”  Several studies

 Please explain what you mean by “Receive MIAMI intervention” under Baseline practice visit 3.  The bullets on the previous page say the patient MIAMI intervention consists of wearing the ABPM and filling out the pre-consultation plan, which are already done at visits 1 and 2, respectively.  This visit sounds like a scheduled HTN-focused visit with their GP.  If that is part of the intervention, it should be included as an additional bullet in the methods description.

 Page 9 1 ^st^ column, this text:  “Analytics such as number of clicks and time spent…” is repeated twice.

Is the study design appropriate for the research question?

Yes

Is the rationale for, and objectives of, the study clearly described?

Yes

Are sufficient details of the methods provided to allow replication by others?

Partly

Are the datasets clearly presented in a useable and accessible format?

Not applicable

Reviewer Expertise:

population health, especially involving chronic disease management for hypertension, diabetes, osteopososis.

I confirm that I have read this submission and believe that I have an appropriate level of expertise to confirm that it is of an acceptable scientific standard.

## References

[1] : Resistant hypertension: an approach to management in primary care. *Journal of Family Medicine and Primary Care* .2015;4(2) : 10.4103/2249-4863.154630 10.4103/2249-4863.154630 PMC440870025949966

# Reviewer report: Reviewer response for version 1

**DOI:** 10.21956/hrbopenres.14939.r34045

**Published:** 2023-07-10

**Authors:** Buna Bhandari

**Affiliations:** 1 University of New South Wales, Sydney, New South Wales, Australia

**MIAMI study**

 This is an interesting study to address the non-adherence to antihypertensive medication and prevent further complications. I have added some comments for the improvement of the quality of the article.

1. Objectives need revision, e.g. objectives 2,3, and 5 do not require “to collect quantitative and qualitative data”. Author can directly write “to assess the feasibility outcomes” and how those objectives will be assessed, “Data collection process” can be detailed in the methods section. Rather it’s better to mention what are those feasibility outcomes if possible.
2. The protocol should be registered in a clinical trial registry such as ISRCTN, and the registration number should be provided in the paper. I noticed it is under process, so please provide the number once registered.
3. Methods - please provide little details about the characteristics of the study cluster. In addition, it’s not compulsory to have power calculation for the sample size in the pilot study, but it is worth mentioning how those 6 clusters and 10 participants in each cluster were decided. Please also specify whether these 10 include only patients or patients and GPs. There is a mismatch in the information provided in the manuscript.
4. I am not clear why the acceptability interview with the control arm is needed; what were the objectives of this interview? Please specify.
5. Table 2 provides details about the MIAMI intervention, which is clear. However, the author has indicated in the background that Behavior change and Collective Intelligence methodologies were used to design the intervention, so it is unclear how those models/theories were used and how the intervention was designed. Though the author plans to discuss that in a separate paper, it is very important to provide some details about the intervention design process (MIAMI booklet, Computer guide development etc.)  to ensure the robustness and comprehensiveness of this intervention.
6. Please provide more details about the SWAT, as it is unclear how the participants will be randomized, whether they will be randomized within each cluster or not. In addition, which objectives of this pilot trial will lead to this SWAT? Need more clarification about the approach; it is confusing to the reader.

Is the study design appropriate for the research question?

Yes

Is the rationale for, and objectives of, the study clearly described?

Yes

Are sufficient details of the methods provided to allow replication by others?

Partly

Are the datasets clearly presented in a useable and accessible format?

Not applicable

Reviewer Expertise:

Implimentation science research, CVD managment, Digital health. Co-design of the intervention, Epidemiology

I confirm that I have read this submission and believe that I have an appropriate level of expertise to confirm that it is of an acceptable scientific standard, however I have significant reservations, as outlined above.

# Response

**Published:** 2023-08-08

**Authors:** Eimear Morrissey

**Affiliations:** University of Galway, Ireland

*This is an interesting study to address the non-adherence to antihypertensive medication and prevent further complications. I have added some comments for the improvement of the quality of the article.*

 We thank the reviewer for taking the time to review our manuscript. The comments have been very useful and we have added our responses below.

1. *Objectives need revision, e.g. objectives 2,3, and 5 do not require “to collect quantitative and qualitative data”. Author can directly write “to assess the feasibility outcomes” and how those objectives will be assessed, “Data collection process” can be detailed in the methods section. Rather it’s better to mention what are those feasibility outcomes if possible.*

We have revised the framing of objectives 2, 3 and 5 in line with this suggestion and agree that the description of objectives is now clearer.

1. *The protocol should be registered in a clinical trial registry such as ISRCTN, and the registration number should be provided in the paper. I noticed it is under process, so please provide the number once registered.*

As noted, the ISRCTN registration was underway when this manuscript was submitted. Registration has been completed and the registration number is ISRCTN85009436. This has been updated in the manuscript (under section heading ‘Aims of the MIAMI cluster pilot RCT).

1. *Methods - please provide little details about the characteristics of the study cluster. In addition, it’s not compulsory to have power calculation for the sample size in the pilot study, but it is worth mentioning how those 6 clusters and 10 participants in each cluster were decided. Please also specify whether these 10 include only patients or patients and GPs. There is a mismatch in the information provided in the manuscript.*

We have added a description of the cluster (under section heading ‘Sample size calculations’)

 As mentioned in the manuscript, the number of clusters chosen was governed by pragmatism and to provide a diverse sample (urban and rural practices) from the population of clusters from which data in the definitive trial will be sampled. Our study statistician has estimated that for any future definitive trial, the population mean systolic clinic BP from this sample will be accurate to within +/- 7 mmHg and the estimated population standard deviation to within 4 units. However, we are very aware that significant caution is required in applying such figures from pilot to definitive trials, and for this reason consider these estimates to be for illustration only.

 The ten participants per cluster in this case refer to patient participants. This is stated in the ‘sample size calculations’ section. The number of GP participants in each cluster will be dependent on practice size.

1. *I am not clear why the acceptability interview with the control arm is needed; what were the objectives of this interview? Please specify.*

The objectives of these interviews are to explore the acceptability of being allocated to the ‘usual care’ arm of a pilot RCT in this context. This is an important aspect to explore, as previous research has shown that study participants can sometimes misunderstand clinical equipoise, and express a preference for intervention arm allocation ^1^.  These interviews will add important data to the assessment of the overall acceptability of conducting a pilot RCT in primary care in Ireland.

 1.         Houghton C, Dowling M, Meskell P, et al. Factors that impact on recruitment to randomised trials in health care: a qualitative evidence synthesis. *Cochrane Database Syst Rev*. 2020;(10).

  

1. *Table 2 provides details about the MIAMI intervention, which is clear. However, the author has indicated in the background that Behavior change and Collective Intelligence methodologies were used to design the intervention, so it is unclear how those models/theories were used and how the intervention was designed. Though the author plans to discuss that in a separate paper, it is very important to provide some details about the intervention design process (MIAMI booklet, Computer guide development etc.)  to ensure the robustness and comprehensiveness of this intervention.*

As the reviewer has noted, we plan to outline the full development process of the MIAMI intervention in a separate paper, which is currently nearing completion. However, we agree that it is useful to include some detail here and we have added this (under section heading’ Introduction’; final paragraph).

1. *Please provide more details about the SWAT, as it is unclear how the participants will be randomized, whether they will be randomized within each cluster or not. In addition, which objectives of this pilot trial will lead to this SWAT? Need more clarification about the approach; it is confusing to the reader.*

Thank you for highlighting this, we have now restructured and added more detail to the SWAT section and believe it is much clearer (under section heading ‘Study within a trial (SWAT)’).
